# Barshop Symposium on Aging 2018 Abstracts

**DOI:** 10.1080/20010001.2019.1591258

**Published:** 2019-03-19

**Authors:** Warren Ladiges

**Affiliations:** Editor-in-Chief

The theme of the 2018 Barshop Symposium on Aging at the Mayan Ranch in Bandera, Texas, USA was ‘Exercise regulation of biological aging’. Drs. Nicolas Musi and Darpan Patel were the conference organizers. The keynote address was delivered by Dr. Charlotte Peterson, Professor and Director of the Center for Muscle Biology at the University of Kentucky, USA, followed by scientific sessions on 1) Effects of exercise on mitochondrial biology; 2) Effects of exercise on inflammation and senescence; 3) Aging, exercise and function; 4) Muscle as a secretory organ; 5) Exercise and metabolism; 6) Exercise and cancer. These platform presentations were complimented by interactive poster presentations, which are represented by respective abstracts for this publication.

## Exercise capacity and physical function in older mice

Ted G. Graber^1,2^, Christopher S. Fry^1^, Rosario Maroto^1^, Blake B. Rasmussen^1,^*

^1^Department of Nutrition and Metabolism, ^2^Division of Rehabilitation Science University of Texas Medical Branch at Galveston (UTMB), TX, USA

In older adults, sarcopenia and frailty produce a downward trajectory of decreased ability to perform activities of daily living, increased incidence of falls, eventual loss of independence, and increased mortality rate. To study the basic mechanisms underlying these diseases, and to assess potential therapies such as exercise, we need to comprehensively evaluate physical ability and exercise capacity in mice as they age. We hypothesized that older C57BL/6 male mice (24/25-month old, n = 30) would have reduced functional/exercise capacity compared to adult mice (6/7-month old, n = 30). We tested this hypothesis with grip test (forelimb strength), rotarod (overall motor function), inverted cling (overall strength/muscle endurance), treadmill (cardiovascular endurance), and voluntary wheel running (volitional exercise/activity rate). We then developed a composite scoring system comprised of the individual functional tests. In subsets from both age groups we determined muscle function using in vivo and in vitro contractile physiology. After adjusting for body mass with univariate ANCOVAs, older mice had reduced capacity in inverted cling (−37%, p < 0.001), grip test (−20%, p = 0.001), rotarod (−29%, p < 0.001), and voluntary wheel running (−72%, p < 0.001). Additionally, maximum dorsiflexion torque (−19%, p = 0.003), and soleus (22%, p < 0.001) and EDL (−37%, p < 0.001) peak force were lower in older mice. The composite scoring system determined reduced physical ability in older mice (p < 0.001) overall. However, older mice performing in the top 25% maintained function, and were indistinguishable from average adult mice (p = 0.335). In conclusion, our composite test is a powerful, non-invasive, and repeatable system to measure exercise capacity and functional aptitude in in future preclinical aging research.

*Correspondence to: Blake B. Rasmussen, UTMB, TX. Email: blrasmus@utmb.edu

##  Funding

NIA P30 AG024832 – Pepper Center Pilot Project (TGG)

## The effect of metformin treatment and voluntary exercise on Alzheimer’s disease related neuropathology in a female transgenic mouse model

A. Jaldi^1^, F. Bello^2^, V. Mulgrave^3^, A. Alsayegh^3^, T. Ajisebutu^2^, T. Falegan^2^, D. Lee^1^, J.S. Allard^1,^*

^1^Department of Physiology & Biophysics, Howard University College of Medicine, Washington, D.C., USA ^2^Department of Biology, Howard University, Washington, D.C., USA ^3^Department of Nutritional Sciences, Howard University, Washington, D.C., USA

Our previous study showed that long-term high-dose treatment with the insulin-sensitizing drug metformin decreased brain-derived neurotrophin (BDNF) levels in mouse brain. However, several studies have shown metformin to be beneficial in the brain, as well as peripherally. In order to attenuate any possible deleterious effects of metformin, we combined a lower-dose metformin treatment with voluntary exercise. The overall goal of this study is to understand the impact of metformin combined with voluntary exercise on amyloid-beta plaque deposition, levels of brain BDNF and pro-inflammatory cytokines using an adult, female APP+/PS1+ mouse model. In cage, voluntary running-wheel activity was monitored and metformin was given at a dose of 70 mg/kg body weight for one year. Preliminary results reveal a significant increase in voluntary running activity of mice treated with metformin. Amyloid-β 42/40 ratio in metformin treated sedentary mice was significantly lower than that of non-treated sedentary mice. The combination of metformin and exercise, however, did not affect amyloid-beta plaque load, levels of mature BDNF or pro-inflammatory cytokines in the hippocampus. These results provide new information on the impact of long-term treatment with metformin in combination with exercise on AD-like pathology.

*Correspondence to: Joanne Allard, Howard University College of Medicine at Washington, D.C. Email: joanne.allard@howard.edu

## Unacylated ghrelin prevents skeletal muscle atrophy and contractile dysfunction in sarcopenia

Bumsoo Ahn^1^, Rojina Ranjit^1^, Holly Van Remmen^1,^*

^1^Department of Aging and Metabolism, Oklahoma Medical Research Foundation at Oklahoma City (OMRF), OK, USA

Sarcopenia, the age-related loss of muscle mass and contractile dysfunction, universally affects the elderly and is closely associated with frailty and reduced healthspan. Despite the inevitable consequences of aging, no pharmacological therapies are available to mitigate sarcopenia. Ghrelin is a gut-released hormone known to increase appetite and body weight through acylation-dependent (acyl ghrelin, AG) receptor activation in pituitary and hypothalamus. Literature shows a direct impact of unacylated ghrelin (UnAG) on skeletal muscle in acute myopathies (e.g. denervation), independent of its receptor activation. However, the impact of UnAG on a progressive loss of muscle mass in sarcopenia has not been tested. Thus, we are testing the hypothesis that UnAG can ameliorate sarcopenia. To test the hypothesis, we used a mouse model of accelerated aging, mice lacking CuZnSOD (Sod1KO). UnAG was continuously administered to two-month old Sod1KO mice for 3 weeks. Serum UnAG level trended to decrease in Sod1KO mice compared with WT, but UnAG increased circulating UnAG level by 2–3 folds. Unlike AG, UnAG did not affect food consumption or body weight. Importantly, the UnAG treatment prevented loss of skeletal muscle mass and contractile dysfunction observed in Sod1KO mice. Our data indicate a direct role of UnAG on sarcopenia independent from food consumption and body weight. These findings implicate UnAG as a potential therapeutic strategy and warrant future aging studies.

*Correspondence to: Holly Van Remmen, OMRF, TX, USA. Email: Holly-VanRemmen@omrf.org

## Ldlr-related protein 1 increases cytokine sensitivity implications for recovery after brain damage

Sadiya Ahmad^1,^*, Shane Sprague^1^, Pamela Reed^1^, Naomi L. Sayre^1^

^1^University of Texas Health Science Center at San Antonio (UTHSCSA), TX, USA

Stroke is the leading cause of death and the primary cause of long-term disability in the U.S. Expression of Apolipoprotein E4 predisposes stroke patients to poor long-term outcome. This study aims to test one possible mechanism by which ApoE4 contributes to cognitive decline after stroke. Here, we examine the effect of a major ApoE4 receptor, low density lipoprotein receptor related protein 1 (LRP1) on sensitivity to stress in astrocytes. LRP1 binds and moves extracellular ligands and plasma membrane proteins into the endocytic system. Others have shown that LRP1 regulates cell-surface TNF receptor (TNFR1) in non-astrocytic cells. We propose LRP1 similarly regulates TNFR1 in astrocytes to attenuate inflammatory response after stroke. Studies have shown ApoE4 slows the recycling of endocytic LDL receptors. We hypothesize that ApoE4 inhibits the ability of LRP1 to remove TNFR1 from the plasma membrane. This is expected to increase cytokine sensitivity, resulting in worse outcome after stroke. We investigated the effect of LRP1 on astrocyte TNFα signaling and response in immortalized ApoE null mouse astrocytes subjected to lentiviral-mediated knockdown of LRP1. We found that LRP1 deficient cells have increased TNFR1 activity and significant loss of viability after prolonged TNFα stimulation. Our results indicate that loss of LRP1 renders astrocytes more sensitive to TNFα. These studies will elucidate how astrocyte-LRP1 contributes to outcome after stroke, and help explain one potential way ApoE4 exerts pathological effects. A better understanding of the long-term processes after stroke will allow identification of therapies which will improve the morbidity and mortality associated with stroke.

*Correspondence to: Sadiya Ahmad, UTHSCSA, TX, USA. Email: ahmedsn@uthscsa.edu

## Aster-b regulates cholesteryl ester trafficking to mitochondria

John-Paul Andersen^1,^*, Kennedy Mdaki^1^, and Yuguang Shi^1^

^1^Barshop Institute for Longevity and Aging Studies, Department of Pharmacology, University of Texas Health Science Center at San Antonio (UTHSCSA), TX, USA

Aster-B also known as GRAMD1B has been characterized as a non-vesicular Plasma Membrane (PM) to Endoplasmic Reticulum (ER) cholesterol transporter. However, little is known as to its fate after it becomes localized in the ER. Here we identified a potential role for Aster-B as a regulator of cholesteryl ester trafficking to the mitochondria as well as a novel function of COPI vesicles in its associated pathway.

*Correspondence to: John-Paul Andersen, UTHSCSA, TX, USA. Email: andersenj@uthscsa.edu

## Linking gut microbiome composition and tlr4-mediated endotoxemia to insulin resistance and type 2 diabetes

Eric Baeuerle^1,^*, Hanyu Liang^1,2^, Nicolas Musi^1,2^

^1^Barshop Institute for Longevity and Aging Studies, University of Texas Health Science Center at San Antonio (UTHSCSA), TX, USA. ^2^Geriatric Research Education and Clinical Center, Audie L. Murphy VA Medical Center, San Antonio, TX, USA

Skeletal muscle insulin resistance is an early-stage dysfunction observed in the pathogenesis of type 2 diabetes (T2DM) in humans. Development of insulin resistance is a multifactorial process between organ systems, and recently, the gut and its microbial contents (the microbiome) have been hypothesized to contribute a larger-than-anticipated role to the development of obesity and type 2 diabetes. These include alteration of the bacterial profile such as reduced Bifidobacterium and Bacteroidetes in the gut, increased intestinal permeability, and production of bacterial lipopolysaccharide (LPS) leading to low-grade systemic inflammation termed endotoxemia. This endotoxemia effect may be primarily mediated through the toll-like receptor TLR4-NF-κB signaling pathway, which is increased in insulin-resistant diseases such as type 2 diabetes. To date, the contributions of the human gut microbiome to endotoxemia and skeletal muscle insulin resistance in obesity and type 2 diabetes have not yet been evaluated. This study will investigate (i) the human gut microbiome profile in lean, obese, and diabetic patients; (ii) endotoxemia modulation in human subjects and its effects on insulin sensitivity and intestinal permeability; and (iii) the effects of endotoxemia and associated inflammation on skeletal muscle insulin resistance in vitro. I hypothesize that alterations in gut microbiome in obese and type 2 diabetic patients result in increased metabolic endotoxemia and systemic inflammation through the TLR4-NF-κB pathway, which contributes to insulin resistance in skeletal muscle tissue. I also hypothesize that the endotoxemia effect can be lessened by promotion of a more favorable microbiome population through synbiotic supplementation or by LPS removal.

*Correspondence to: Eric Baeuerle, UTHSCSA, TX, USA. Email: baeuerle@livemail.uthscsa.edu

## Investigating the loss of terminal neuronal differentiation as a novel mechanism driving neuronal death in Alzheimer’s disease and related tauopathies

Adrian Beckmann^1,^*, Habil Zare^2^, Bess Frost^3^

^1^Institute of Biotechnology, Barshop Institute of Longevity and Aging, UTHSCSA, TX, USA. ^2^Department of Computer Science, Texas State University at San Antonio, TX, USA. ^3^Department of Cell Systems and Anatomy, Barshop Institute of Longevity and Aging, University of Texas Health Science Center at San Antonio, TX, USA

Tauopathies are a class of neurodegenerative disorders associated with deposits of insoluble tau protein within the brain. At over 5 million cases currently diagnosed among Americans, Alzheimer’s disease (AD) is the most common type of tauopathy. As one of two hallmark pathologies of AD, pathogenic tau has emerged as a promising target for therapeutic targeting. Mutations in the tau gene are associated with dominantly inherited familial tauopathies termed frontotemporal dementia with parkinsonism linked to chromosome 17 (FTDP-17), demonstrating that tau dysfunction is sufficient to drive neurodegeneration. An active cellular program maintains the terminally differentiated, postmitotic state of neurons. When this program is perturbed, post-mitotic neurons can re- activate the cell cycle, which is known to drive neuronal death. In my preliminary analysis, I have identified prospero, a protein that orchestrate the expression and silencing of genes that maintain terminal neuronal differentiation, among the top ten significantly downregulated genes in brains of a Drosophila model of tauopathy. In addition, I identified 51 genes that are differentially expressed in tau transgenic Drosophila that are known to be regulated by prospero, suggesting that pathogenic tau may disrupt the cellular program that maintains terminal neuronal differentiation. Therefore, I hypothesize that pathological tau causes neuronal death by dysregulating prospero and staufen, thereby disrupting the cellular program that maintains terminal differentiation in neurons. Using genetic manipulations of tau transgenic and control Drosophila, I have identified filamentous actin to be a key driver in the depletion of prospero and discovered that targets of prospero affect tau-induced neurotoxicity.

*Correspondence to: Adrian Beckmann, Institute of Biotechnology, Barshop Institute, UTHSCSA, TX, USA. Email: beckmann@livemail.uthscsa.edu

## 4-phenylbutyrate: effects on dj-1/park7 and potential as an aging intervention

Michael Bene^1^, Adam Salmon^1,^*

^1^University of Texas Health Science Center at San Antonio, TX, USA

4-Phenylbutyrate (PBA), an FDA approved drug for urea cycle disorders, has been extensively researched for its potential use in many age-related diseases including: Alzheimer’s, Parkinson’s, type II diabetes, heart disease, and various cancers. One gene whose expression has been reported to increase in response to PBA is the Parkinson’s related oxidative stress response gene DJ-1/PARK7. DJ-1 promotes cell survival pathways and protects mitochondrial function. Most research looking at PBA’s effects on DJ1 have concentrated on neurons, therefore PBA’s effects on DJ-1 in other cell types are largely unknown. To address this question dose response experiments were performed in vitro with mouse liver cells, C2C12 muscle cells, and tail fibroblasts. DJ-1 protein levels were increased in response to PBA in both liver cells and tail fibroblasts, but not C2C12 cells. Notably, tail fibroblasts from a male mouse had significantly different DJ-1 levels compared to fibroblasts taken from a female mouse. Additionally, due to DJ-1’s ability to complex with PINK1/Parkin and mitochondrial complex I, it was expected that PBA may be altering mitochondrial function through DJ-1. Interestingly, it was found that despite no alteration to DJ-1 expression, PBA produced changes to mitochondrial respiration in C2C12 cells, reducing basal respiration and relative ATP production, while leaving relative maximum respiration unchanged. PBA has been approved by the NIA Intervention Testing Program to determine possible effects on mammalian lifespan and future in vivo studies may further elucidate the role of PBA in altering physiological function and determine possible effectiveness in a broader range of patients.

*Correspondence to: Adam Salmon, Barshop Institute, UTHSCSA, TX, USA. Email: salmona@uthscsa.edu

## Oxidative stress is associated with muscle damage without a myogenic response

Jacob L. Brown^1^, Bumsoo Ahn^1^, Gavin Pharaoh^1^, Ashwini Poopal^1^, Rojina Ranjit^1^, Holly Van Remmen^1,^*

^1^Aging and Metabolism Research Program, OMRF, Oklahoma City, OK, USA

Oxidative stress is associated with sarcopenia; however, the effect of oxidative stress on myogenesis is unknown. Therefore, the purpose of this study was to examine myogenic factors in inducible MnSOD muscle specific knockout (Sod2 mKO) mice. Methods: Skeletal muscle Sod2 was deleted with Cre recombinase containing mutated estrogen receptor ligand-binding domains driven by α-actin promoter. Wildtype (WT) or Sod2 floxed mice were injected with tamoxifen at ~6 months old to delete the floxed gene and were sacrificed at 11–13 months old. Sod2 protein and activity level were measured to confirm deletion of the transgene. Muscle wet weights, contractility, respiration and reactive oxygen species (ROS) were also measured. Hematoxylin and eosin staining was used to assess muscle size and morphology. Myogenic markers were measured by rt-qPCR and/or western blot. Data were analyzed by t-test, and α was set at 0.05. Results: Sod2 was not detectable in Sod2 mKO muscle. Muscle size, respiration, ROS and contractility were not different between groups; however, there was a mean increase in ROS. Centrally located nuclei were ~10 fold higher in Sod2 mKO mice, so myogenic response was assessed. MyoD mRNA content and Myogenin protein content were measured but were found to be not different between groups; however, eMHC mRNA content was ~50% lower in Sod2 mKO mice. Conclusions: Centrally located nuclei is associated with regenerating fibers; however, myogenic response was lacking. This may indicate satellite cell dysfunction. Satellite cell response to muscle damage will be assessed in future studies.

*Correspondence to: Holly Van Remmen, OMRF, TX, USA. Email: Holly-VanRemmen@omrf.org

## Effect of sex on age-specific mortality reduction in response to anti-aging interventions in genetically heterogeneous mice

Catherine J. Cheng^1,^*, Qianqian Liu^1^, James F. Nelson^1^, Jonathan A.L. Gelfond^1^

^1^University of Texas Health Science Center at San Antonio (UTHSCSA), TX, USA

The female survival advantage is one of the most robust characteristics of human longevity, but the underlying biological mechanisms are unknown. The National Institute on Aging’s Interventions Testing Program (ITP) was designed to overcome the limitations of inbred strains by evaluating lifespan-extending compounds in genetically heterogeneous UMHET3 mice. In this combined dataset of 12,223 UM-HET3 mice from the ITP, we report that the female survival advantage parallels that of human populations: greatest in early adulthood and diminishing progressively thereafter. This finding is observed at 3 sites in 6 cohort years. We further report that, out of 13 lifespan-extending interventions tested, those with a male-specific effect primarily decrease male mortality before median lifespan, whereas interventions that were effective in both sexes are more likely to reduce mortality at later ages (>30 months).

*Correspondence to: Catherine J Cheng, UTHSCSA, TX, USA. Email: ChengCJ@livemail.uthscsa.edu

## The adapted endoplasmic reticulum (ER)-associated protein quality control (ERQC) is critical for the long-lived Caenorhabditis elegans rpn-10 proteasome mutant

Meghna N. Chinchankar^1,2,3,^*, Karl A. Rodriguez^1,4^, Alfred L. Fisher^1,2,3,5^

^1^Barshop Institute for Longevity and Aging Studies, ^2^Center for Healthy Aging, ^3^Division of Geriatrics, Gerontology, and Palliative Medicine, Department of Medicine, ^4^Department of Cell Systems and Anatomy, UTHSCSA, San Antonio, TX, USA. ^5^Division of Geriatrics, Gerontology, and Palliative Medicine, Department of Medicine, University of Nebraska Medical Center, Omaha, NE, USA

Protein degradation is essential for the preservation of the proteome. Its reduced efficiency leads to protein aggregation which potentiates several proteotoxic disorders. Paradoxically, our lab reported that the Caenorhabditis elegans rpn-10(ok1865) proteasome mutant exhibits enhanced proteostasis, elevated stress resistance and extended lifespan. RPN-10 is an ubiquitin receptor on the 19S regulatory particle that targets polyubiquitinated substrates to its 20S proteasomal core for degradation. The proteasome dysfunction of the rpn-10 mutant is characterized by reduced ubiquitin fusion degradation. We ascertained that the compensatory upregulation of autophagy and SKN-1/Nrf-mediated responses partially contribute to the robust rpn-10 mutant phenotype. To elucidate this protective mechanism, our RNA-sequencing data analysis revealed that several ERQC genes are transcriptionally upregulated in the rpn-10 mutant. We hypothesized that the rpn-10 mutant exhibits enhanced ER proteostasis which mediates its stress resistance. Accordingly, we found that cytosolic proteostasis and longevity depends on the ER master chaperone hsp-3/-4 (BiP) and ER ATPase cdc-48.2 (p97/VCP) in the rpn-10 mutant. Moreover, it exhibits altered ER homeostasis compared to the wild-type. Complementarily, the attenuated expression of the aggregation-prone mutant α-1 antitrypsin (ATZ) reporter proves that ER proteostasis is ameliorated in the rpn-10 mutant. Via a suppressor screen of the ATZ phenotype in the rpn-10 mutant, we identified H04D03.3 (putative homolog of the proteasome-associated protein ECM29) is critical, thus signifying an unexpected role for the rpn-10 mutant proteasome in maintaining ER proteostasis. Altogether, it appears that mild proteasomal dysfunction induces an ERQC adaptation that underlies the improved proteostasis and increased longevity of the rpn-10 mutant.

*Correspondence to: Meghna N. Chinchankar, Barshop Institute, UTHSCSA, TX, USA. Email: Chinchankar@livemail.uthscsa.edu

## Mitochondrial thioredoxin reductase 2 overexpression enhances glucose metabolism in mice

E. Sandra Chocron^1^, Ning Zhang^1^, Nicolas Musi^1,2^, Andrew M. Pickering^1,3,^*

^1^Barshop institute for Longevity and Aging Studies, UT Health San Antonio, TX, USA. ^2^Geriatric Research, Education, and Clinical Center (GRECC), South Texas Veterans Health Care System (STVHCS) at San Antonio, TX, USA. ^3^Department of Molecular Medicine, School of Medicine, UT Health San Antonio, TX, USA

Mitochondrial Thioredoxin Reductase (Txnrd2) is a rate limiting enzyme in the mitochondrial thioredoxin system which serves as one of the major mitochondrial ROS scavenging pathways. Txnrd2 is also a repressor of the ASK-1 oxidative stress induced apoptotic pathway. Our group previously identified a correlation with the expression of this protein and long-lived species and its overexpression prolonged lifespan in Drosophila. We have generated a Txnrd2 transgenic (T-tg) mouse which has ubiquitously heighted (two-fold) Txnrd2 expression. We have found that overexpression of Txnrd2 leads to increased Oxygen Consumption, enhanced mitochondrial membrane potential and increased resistance to mitochondrial oxidative damage in MEFs. We have also found that female T-tg mice showed a leaner trend and reduced food consumption, with improved glucose tolerance but no difference in insulin sensitivity. These mice showed a lower Oxygen consumption and CO2 production with lower energy expenditure in individual metabolic cages. We further tested their exercise capacity where the T-tg mice had a similar performance to control mice. These results suggest that Txnrd2 overexpression can lead to metabolic changes that need to be further understood, but seem to be beneficial for glucose handling and homeostasis in female mice.

*Correspondence to: Andrew M. Pickering, Barshop Institute, UTHCSA, TX, USA. Email: PickeringAM@uthscsa.edu

##  Funding agency

Voelcker foundation.

## The effect of late life β-gpa supplementation on age related declines in het3 mice

Dorigatti JD^1,^*, Liu Y^1^, Thyne KM1, Salmon AB^1^

^1^The Sam and Ann Barshop Institute for Longevity and Aging Studies, UTHCSA, San Antonio, TX, USA

β-Guanidinopropionic acid (β-GPA) is a naturally occurring creatine analog reported to activate AMP Activated Protein Kinase (AMPK) signaling. β-GPA has been reported to improve glucose metabolism, promote mitochondrial biogenesis, alter mitochondrial fuel preference, and enhance muscle exercise resistance in young animals. However, there is a knowledge gap regarding whether β-GPA may improve or prevent age-related declines in such physiological measures when delivered later in life. We consequently hypothesize that β-GPA will prevent age-related physiological declines in function by altering mitochondrial energetics to preserve cellular function. We test this hypothesis using young and old genetically heterogeneous mice (HET3) fed 1% β-GPA or control chow for 4 months. In contrast to reports of decreased body mass in young rodents, we report that aged males supplemented with β-GPA are protected from late-life declines in body mass. Further, while young rodents are reported to exhibit improved exercise tolerance and better glucose tolerance with β-GPA, aged mice did not see improvements in these measures, though HbA1c levels were decreased in males. Unexpectedly, content of mitochondrial oxidative phosphorylation complexes was generally unchanged by β-GPA, while Complex 1 was reduced in β-GPA treated males. Additionally, Acetyl Coenzyme A Carboxylase (ACC) phosphorylation was increased in females supplemented with β-GPA but no corresponding increase in fatty acid oxidation was observed in permeabilized soleus. These results raise an important point regarding the timing of interventions to improve healthy aging; while some interventions may have rejuvenating effects in the old, others may require earlier intervention to provide benefit.

*Correspondence to: Jonathan Dorigatti, Barshop Institute, UTHSCSA, TX, USA. Email: dorigatti@uthscsa.edu

## Activation of SF-1 neurons induces PGC-1α in the skeletal muscle

Takuya Yoshida^1,2^, Nancy S. Gonzalez^2^, Teppei Fujikawa^2,3,4,^*

^1^Department of Clinical Nutrition, School of Food and Nutritional Sciences, University of Shizuoka, Shizuoka, Japan. ^2^Department of Cellular and Integrative Physiology, Long School of Medicine, ^3^Center for Biomedical Neuroscience, ^4^Mouse Genome Engineering and Transgenic Facility, Long School of Medicine, UTHSCSA, TX, USA

Exercise training induces metabolic adaptations such as strengthening skeletal muscle function, leading to beneficial effects of exercise on metabolism. However, the precise mechanism underlying metabolic adaptations to exercise, in particular, the contribution of the central nervous system (CNS), is largely unknown. Our previous study demonstrates that steroidogenic factor-1 (SF-1) in the ventromedial hypothalamic nucleus (VMH) is key to the metabolic adaptions to exercise. Here we investigate that the role of neuronal activities of SF-1-expressing neurons (SF-1 neurons) in the regulation of metabolic adaptations. We utilized optogenetics which allows us to specifically activate SF-1 neurons. After SF-1 neuronal activation by optogenetics, we measured mRNA levels of peroxisome proliferator-activated receptor gamma coactivator 1-alpha (PGC-1α), which is the master regulator of skeletal muscle function as well as one of readouts of exercise effects to the skeletal muscle. We found that activation of SF-1 neurons is sufficient to increase PGC-1α in the skeletal muscle. We further found that blocking sympathetic nervous system (SNS) blunted increased PGC1α in the skeletal muscle by the activation of SF-1 neurons. Overall, our data suggest that the activation of SF-1 neurons-SNS axis is sufficient to recapitulate exerciseinduced PGC-1α in the skeletal muscle.

*Correspondence to: Teppei Fujikawa, UTHSCSA, TX, USA. Email: fujikawa@uthscsa.edu

##  Funding Support

This study was supported by UT System Rising STARs Program (to T.F.) and San Antonio Area Foundation Research Grant (to T.F.)

## Reversing age-related decline using non-cytotoxic transplantation of young hematopoietic stem cells

Michael Guderyon^1^, Cang Chen^1^, Anindita Bhattachcharjee^1^, Xiaogang Wang^1^, Guo Xiaoqian^1^, Guo Ge^1^, Senlin Li^1^

^1^Department of Medicine, Biology of Aging, UTHSCSA, TX, USA

Hematopoietic stem cell transplantation (HSCT) offers an effective treatment modality for patients diagnosed with a number of diseases of the blood and immune systems. HSCT uses mobilization factors to collect donor cells before patients undergo toxic conditioning regimens using chemotherapy and/or radiation. Here, we employ a novel non-cytotoxic conditioning regime using FDA-approved mobilization factors in concert with multiple cycles of transplantation to examine health-associated benefits of transplanting youthful donor HSCs into old recipients. We report that mobilization of endogenous hematopoietic stem cells (HSCs) using G-CSF and AMD3100 sufficiently conditions recipients to accept donor cells, achieving over 70% long-term engraftment in C57BL6/NIA mice. In ongoing studies, we define health-associated benefits of replacing old endogenous HSCs with young donor cells by examining healthspan markers such as food intake, body weight, median and overall longevity, and the accumulation of health-associated deficits. This approach is particularly attractive because, in addition to eliminating the need for irradiation or chemotherapy, each of the essential reagents have previously been approved by the FDA for human use. Thus, these studies have the potential to improve current transplantation regimes by replacing traditional HSCT methods with this novel approach and may lead to the development of the first cell-based anti-aging therapy.

*Correspondence to: Michael Guderyon, University of Texas Health Science Center at San Antonio. Email: Guderyon@uthscsa.edu

## Deficiency of a novel depalmitoylase exacerbates diet-induced obesity and insulin resistance

Jason Hadley^1,^*

^1^Biology of Aging, UTHSCSA, TX, USA

Protein palmitoylation is a reversible post-translational modification. It has gained a rapid attention recently as its ability to spatially and temporally modulate a diverse range of signaling pathways in cells. Depalmitoylases are a class of enzymes that catalyze the removal of this lipid modification and subsequently alters the subcellular localization of the target protein. Recently, we identified LF8 as a potential novel depalmitoylase and found its positive association with insulin sensitivity in mouse liver. In addition, we have generated LF8 knockout mice, and our preliminary data indicate that deletion of LF8 gene led to exacerbate high fat diet-induced obesity phenotype. These results reveal an important role of depalmitoylation in regulating energy homeostasis and identify a novel target molecule for enhancing insulin sensitivity and treating type 2 diabetes.

*Correspondence to: Hadley Jason, University of Texas Health Science Center at San Antonio. Email: hadleyj@livemail.uthscsa.edu

## Modeling prion-like tau spread in drosophila melanogaster

Simon A. Levy^1,^*, Bess Frost^2^

^1^Neuroscience discipline, IBMS PhD program, Graduate School of Biomedical Sciences, UTHSCSA, TX, USA. ^2^Department of Cell Systems and Anatomy, UTHSCSA, TX, USA

Tauopathies are neurodegenerative diseases characterized by fibrillar tau protein aggregates. In Alzheimer’s disease these aggregates form neurofibrillary tangles, one of the disease’s pathological hallmarks. Tau pathology propagates through the brain hierarchically over time and along circuitry, suggesting a prion-like cell-to-cell mode of spreading. Tau is now known to spread between cells and form stably transmitted strains in vitro and in vivo, but the underlying mechanisms have not been well characterized. We are creating a Drosophila model to study prion-like tau propagation in a shortlived animal model amenable to screening approaches. We aim to model two key features of prionlike tau transmission: 1) cell-to-cell spread and 2) a capacity to recruit soluble tau into aggregates. Native gel electrophoresis indicates that tauP301L forms oligomers in Drosophila neurons in vivo, suggesting a capacity for aggregation. Based on this finding, tauP301L was expressed in retinal neurons and its spread was assessed by fluorescence in situ hybridization to detect tau mRNA and immunofluorescence to detect tau protein. Tau protein was found in cells lacking tau mRNA, indicating cell-to-cell tau spread. To detect aggregation seeding of soluble tau by spreading tau, seedcompetent tauP301L fused to N-terminal luciferase (LucN), will be expressed in one set of neurons, while wild-type tau fused to C-terminal luciferase (LucC) will be expressed in another. LucN-labeled tau recruiting soluble LucC-tagged tau into aggregates will produce luminescence. This model will allow investigations into the mechanisms controlling tau spread in adult brains, with the goal of developing treatment strategies targeting prion-like pathogenic tau spread.

*Correspondence to: Simon A. Levy, UTHSCSA, TX. Email: levys@livemail.uthscsa.edu

## The dopamine metabolite 3,4-dihydroxyphenylacetaldehyde exacerbates motor deficits in an alpha-synuclein mouse model of Parkinson’s disease

Paul Anthony Martinez^1,2,3^, Vanessa Martinez^2^, Elizabeth Fernandez^1,2,4^, Randy Strong^1,2,3,4^

^1^Department of Pharmacology, ^2^Barshop Institute for Longevity and Aging Studies, ^3^Center for Biomedical Neuroscience, UTHSCSA, TX, USA. ^4^Geriatric Research, Education and Clinical Center, South Texas Veterans Health Care Network (STVHCS) at San Antonio, TX, USA

Parkinson’s disease (PD) is second most common neurodegenerative disease affecting ~1–2% of individuals over the age of 65 years. Neurodegeneration of dopaminergic neurons in the nigrostriatal pathway results in a reduction of striatal dopamine (DA) that is causally related to impaired motor function. A pathological hallmark of PD, is the presence of alpha-synuclein (αSyn)-rich Lewy bodies in surviving DA neurons. While it remains unclear what mechanism(s) underlies the selective vulnerability of DA neurons, a growing body of evidence implicate the dopamine metabolite, 3,4-dihydroxyphenylacetaldehyde (DOPAL). DOPAL is the first product of dopamine catabolism by the enzyme monoamine oxidase. We previously reported that deletion of the two aldehyde dehydrogenase (ALDH) enzymes (Aldh1a1 and Aldh2) that are responsible for the detoxification of DOPAL, results in elevated levels of DOPAL, degeneration of tyrosine hydroxylaseimmunoreactive neurons and deficits in motor performance. Additionally, mutations or multiplication of the SNCA gene which encodes for the principal component of Lewy bodies, αSyn, implicates αSyn in PD. Evidence suggests αSyn undergoes aggregation to form neurotoxic oligomers. DOPAL promotes and stabilize αSyn oligomers. The selective generation of DOPAL in DAergic neurons and its ability to stabilize neurotoxic αSyn oligomers, led us to hypothesize that, αSyn is mechanistically related to the neurochemical and behavioral manifestations of PD that result from elevated DOPAL. To test this hypothesis, we generated mice deficient in Aldh1a1 and Aldh2 crossed them to mice that overexpress the human wildtype αSyn. We found that overexpression of αSyn in the presence of elevated levels of DOPAL was associated with exacerbation of deficits in motor performance. The results of neurochemical assays will be discussed.

*Correspondence to: Anthony Martinez, Barshop Institute, UTHSCSA, TX, USA. Email: martinezp3@uthscsa.edu

##  Funding

VA/BLR&D (I01 BX001641) and NIH/NIA-Nathan Shock Center of Excellence in Basic Biology of Aging (P30-AG013319)

## Apolipoprotein E genotype modulation of the impact of resveratrol and exercise training on brain and muscle function

Verona Mulgrave^1^, Abdulrahman Alsayegh^1^, Aida Jaldi^1^, Joanne Allard^1,^*

^1^Department of Physiology & Biophysics, Howard University College of Medicine, Washington, D.C, USA.

Possession of the APOE e4 allele has been associated with a higher risk for developing Alzheimer’s disease. Recent studies have suggested that ApoEε4 (ε4) down-regulates expression of brain-derived neurotrophic factor (BDNF) a critical promoter of synaptic plasticity and neuron survival. Upregulation of BDNF via aerobic activity is well documented and aerobic exercise has been shown to improve cognitive function. Our recent pilot study on elderly African Americans showed a disparity in exercise-induced BDNF upregulation between ε4 and ε3 carriers. Through this study we strive to assess the impact of APOE variance on exercise capacity and exercise induced regulation of biomarkers of brain health and function. C57Bl6, APOE ε3 add APOEε4 mice were placed in exercise and sedentary groups. Baseline locomotor function was measured using rotarod training and in-cage running-wheel activity. Mice in exercise groups were subjected to 30 minutes of treadmill running, 5days a week for 8 weeks. Voluntary activity in C57Bl6 mice was significantly greater than ε3 and ε4 mice. In turn, ε3 mice were significantly more active than ε4 mice. Despite activity level differences, hippocampal levels of mature BDNF were similar. Various blood cytokine and lipid levels varied between genotype and activity groups. Exercise activity conferred variable effects on activity and blood biochemistry in APOE variant mice.

*Correspondence to: Joanne Allard, Howard University College of Medicine at Washington, D.C. Email: joanne.allard@howard.edu

## Proteasome function in Alzheimer’s disease

Erin Munkácsy^1,^*, Laura Quintanilla^1^, Andrew M. Pickering^1^

^1^Barshop institute of Aging Studies and Longevity, UTHSCSA, TX, USA

The proteasome has key roles in neuronal proteostasis, including protein turnover, removal of misfolded or oxidized proteins, presynaptic protein turnover, synaptic efficacy, and synaptic plasticity. Proteasome dysfunction occurs in the brain under Alzheimer’s disease (AD) and related dementias and there is strong evidence that β-amyloid (Aβ) inhibits proteasome function. We show in Drosophila models of AD that proteasome depletion accelerates, while proteasome augmentation delays, AD-like pathology and cognitive deficits. Surprisingly, Aβ accumulation was not reduced by proteasome upregulation, suggesting that proteasome dysfunction triggered by Aβ inhibition drives downstream neurodegeneration rather than altering Aβ accumulation.

*Correspondence to: Erin Munkácsy, Barshop Institute, UTHSCSA, TX, USA. Email: Munkacsy@uthscsa.edu

## Tau-induced astrocyte senescence as a driver of neuronal dysfunction in Alzheimer’s disease

Angela Olson^1,2^, Stacy Hussong^1,2,3,^*, Rakez Kayed^4^, Veronica Galvan^1,2,3^

^1^Department of Cellular & Integrative Physiology, ^2^Barshop Institute for Longevity and Aging Studies, UTHSCSA, TX, USA. ^3^STVHCS at San Antonio, TX, USA. ^4^Department of Neurology, UTMB, TX, USA

The accumulation of molecular damage in somatic cells can trigger cellular senescence, an irreversible state of cell cycle arrest accompanied by the expression of proinflammatory mediators known collectively as the ‘senescent-associated secretory phenotype’ (SASP). During the pathogenesis of Alzheimer’s disease (AD) and other tauopathies, the microtubule-stabilizing factor tau is phosphorylated, becomes misfolded, and detaches from microtubules, destabilizing the microtubule cytoskeleton. Misfolded tau forms soluble aggregates that are released extracellularly and are transmitted transneuronally, promoting native tau phosphorylation and aggregation in target cells. We recently showed that, in addition to neurons, soluble extracellular tau aggregates propagate to brain microvascular endothelial cells, where microtubule destabilization triggers senescence/SASP. Neuronally-originated extracellular tau can also reach nearby astrocytes, which express abundant tau. Markers of cellular senescence and inflammation are increased in AD brain, including in astrocytes, and we had previously found that AD tauopathy models also display markers of senescence. In the present study, we tested the central hypothesis that soluble aggregated tau propagates to astrocytes and induces astrocyte senescence/SASP. We found that soluble aggregated tau propagates into primary human astrocytes, and that tau transmission to astrocytes triggered endogenous tau phosphorylation and microtubule destabilization, followed by upregulation of markers of permanent cell cycle arrest and the acquisition of SASP. These data provide the first evidence that soluble aggregated tau propagates to astrocytes and indicate that tau aggregate propagation triggers astrocyte senescence/SASP. We further found that primary neurons cocultured with astrocytes undergoing tau-induced senescence show reduced dendritic arborization and dendritic spine density, suggesting that astrocyte senescence may contribute to synaptic dysfunction in AD. Drugs that eliminate senescent cells are FDA-approved and antibody-based approaches to remove tau from brain are already in clinical trials. Our studies may thus advance the implementation of these interventions for the treatment of AD and other tauopathies.

*Correspondence to: Stacy Hussong, Barshop Institute, UTHSCSA, TX, USA. Email: hussong@uthscsa.edu

##  Funding

NIA T32 AG021890; NIA R01 AG057964-01

## Senolytic treatment rescues tau-associated myelin defects

Alec Peterson^1^, Joseph M. Valentine^2^, Kathryn R. Sickora^2^, Ashley Zapata^2^, Eric Baeuerle^1,2^, Qiang Shen^3^, Miranda E. Orr^1,2,4,^*

^1^San Antonio Geriatric Research, Education and Clinical Center, STVHCS at San Antonio, TX, USA. ^2^Barshop Institute for Longevity and Aging Studies, UTHSCSA, TX, USA. ^3^Research Imaging Institute, University of Texas Health Science Center San Antonio at San Antonio, TX, USA. ^4^Glenn Biggs Institute for Alzheimer’s & Neurodegenerative Diseases at San Antonio, TX, USA

Senescent cell accumulation is associated with tissue pathology, functional decline and decreased lifespan. In the brain, post-mitotic neurons containing aggregated tau in the form of neurofibrillary tangles (NFTs) are a source of cellular senescence. We are investigating the effects of NFT-associated cellular senescence on brain structure and function. Target pathways of interest were derived from studies on postmortem human brain tissue from patients with Alzheimer’s disease and progressive supranuclear palsy. Cellular and molecular pathways disrupted by neuronal senescence were identified by comparing laser capture micro-dissected neurons with NFTs to neurons without NFTs. Transgenic tau mouse models were utilized to investigate specific disease mechanisms responsible for observations in human tissue. Pharmacological interventions were tested in transgenic mice to investigate therapeutic potential of disrupting candidate pathways. Treatment efficacy was evaluated by measuring translationally relevant outcomes (i.e., brain MRI and postmortem histopathology). Neurons with NFTs differentially regulate genes encoding for extracellular matrix and growth factor proteins that alter glial cell biology. Transgenic mouse models acquire pathologies involving all glial cell types, including oligodendrocytes. Analyses indicate tau-associated aberrant myelin gene and protein expression, and NFT-associated white matter hyperintensity (WMH) pathology. Pharmacological removal of senescent cells rescues myelin-associated pathologies in transgenic mice with advanced disease. Tau-induced cellular senescence contributes to WMH pathology and oligodendroglia dysfunction. Aberrant myelin protein expression and WMH pathology is sensitive to senolytic treatment. White matter pathologies, specifically WMH, are associated with cognitive decline and occur in 89% of patients with Alzheimer’s disease; treatment with senolytics may provide benefit to these patients.

*Correspondence to: Miranda Orr, Barshop Institute, UTHSCSA, TX, USA. Email: orrm3@uthscsa.edu

## Multi-tissue epigenetic clocks for mice to quantify aging precisely

Anand Gopalan^1^, Paolo Piatti^1^, Wei Guo^1^, Jessica Valadez^1^, Jae-Hyun Yang^2^, Joe Ollar^1^, Yap Chew^1^, David Sinclair^2,^*, Xiaojing Yang^1^

^1^Zymo Research Corp at Irvine, CA, USA. ^2^Department of Genetics, Harvard Medical School at Boston, MA, USA

Age is a driving factor of many major diseases including cancer, cardiovascular disease, arthritis, osteoporosis and type 2 diabetes. Recent studies have shown that epigenetic changes, especially DNA methylation, are aging related processes. Notably, we have developed a Human DNAge™ test based on Steve Horvath’s epigenetic clock. DNAge™ has been recognized as a robust and precise tool for the determination of biological age, and the study of the mechanisms underlying agerelated disease. Considering the invaluable power of mice as a primary research model, we developed Mouse DNAge™, a murine DNA methylation panel targeting over 2000 epigenomics loci. Mouse DNAge™ takes advantage of our proven targeted bisulfite sequencing approach, called SWARM™ (Simplified Whole-panel Amplification Reaction Method), to accurately quantify and track the epigenetic aging of mice. Epigenetic profiles have been shown to be tissue-specific and therefore, we further built tissue-specific epigenetic clocks for murine whole blood, muscle, liver and brain. To this end, we used the elastic net regression of DNA methylation levels of the targeted loci and the chronological age of over 200 tissue samples collected from mice between 9 and 129 weeks old. Using our Mouse DNAge™ blood clock, we demonstrated the relationship between DNAge™ rate of acceleration and life span amongst different mice strains. Interestingly, we also discovered that only 15% of aging-associated loci overlap between Mouse DNAge™ blood and brain clocks. This demonstrates the importance of tissue-specific clocks for the study of aging mechanisms and intervention strategies targeting specific mice tissues and organs.

*Correspondence to: David Sinclair, Department of Genetics, Harvard Medical School at Boston, MA, USA. Email: david_sinclair@hms.harvard.edu

## Identification of a novel adipokine tetranectin and its metabolic function

George Plasko^1^, Sija He^1^, Jingjing Zhang^2^, Fen Liu^2^, Juli Bai^1,2^, Lily Dong^3^, Feng Liu^1,2^

^1^Department of Pharmacology, UTHSCSA, TX, USA. ^2^Department of Metabolism and Endocrinology, Metabolic Syndrome Research Center, Second Xiangya Hospital, Central South University, Changsha, Hunan, China. ^3^Department of Cell Systems & Anatomy, UTHSCSA, TX, USA

Obesity has an established association with insulin resistance, type 2 diabetes mellitus, dyslipidemia, and atherosclerosis, with a heavy burden on the healthcare system. Preventing the progression from obesity to insulin resistance requires an understanding of the regulatory mechanisms involved in the loss of insulin sensitivity. Adipose tissue is well known to function as an endocrine organ that produces many kinds of adipokines, such as leptin, adiponectin, resistin, and retinol-binding protein 4 etc. In the current study, we report the identification and initial characterization of a novel adipokine tetranectin. Tetranectin, which is coded by the C-type lectin domain family 3 member B (CLEC3B) gene, is ubiquitously expressed in various mouse tissues, whereas it is highly enriched in white adipose tissue. We found that the serum level of tetranectin was much higher in both obese and diabetic patients. Knocking out the tetranectin gene in mice protected against high fat diet-induced insulin resistance and glucose intolerance without effects on food intake and body weight. Mechanistically, tetranectin targets liver tissues and its deficiency improves insulin-stimulated AKT phosphorylation at serine 473. Taken together, we have identified a novel adipokine which mediates metabolic crosstalk among tissues to maintain systemic insulin sensitivity. Further investigation of tetranectin’s function could yield a new target for the development of effective therapeutic treatment for obesity and its associated metabolic diseases.

*Correspondence to: Feng Liu, UTHSCSA, TX, USA. Email: liuf@uthscsa.edu

## Restoration of SERCA ATPase prevents oxidative stress-related muscle atrophy and weakness

Rizwan Qaisar^1^, Shylesh Bhaskaran^1^, Rojina Ranjit^1^, Kavithalakshmi Sataranatarajan^1^, Pavithra Premkumar^1^, Kendra Huseman^1^, Holly Van Remmen^1,2,^*

^1^Aging and Metabolism Research Program, OMRF, TX, USA. ^2^Oklahoma City VA Medical Center, Oklahoma City, OK, USA

Molecular targets to reduce muscle weakness and atrophy due to oxidative stress have been elusive. Here we show that activation of Sarcoplasmic Reticulum (SR) Ca2+ ATPase (SERCA) with CDN1163, a novel small molecule allosteric SERCA activator, ameliorates the muscle impairment in the CuZnSOD deficient (Sod1-/-) mouse model of oxidative stress. Sod1-/- mice are characterized by reduced SERCA activity, muscle weakness and atrophy, increased oxidative stress and mitochondrial dysfunction. 7 weeks of CDN1163 treatment completely restored SERCA activity and reversed the 23% reduction in gastrocnemius mass and 22% reduction in specific force in untreated Sod1-/- versus wild type mice. These changes were accompanied by restoration of autophagy protein markers to the levels in wild-type mice. CDN1163 also reversed the increase in mitochondrial ROS generation and oxidative damage in muscle tissue from Sod1-/- mice. Taken together our findings suggest that the pharmacological restoration of SERCA is a promising therapeutic approach to counter oxidative stress associated muscle impairment.

*Correspondence to: Holly Van Remmen, OMRF, TX, USA. Email: Holly-VanRemmen@omrf.org

## Methionine redox in methionine restriction

Thyne KM^1,^*, Liu Y^1^, Salmon AB^1^

^1^The Sam and Ann Barshop Institute for Aging and Longevity Studies Barshop Institute, UTHSCSA, TX, USA

Caloric restriction (CR) is perhaps the most well-studied and robust method for improving lifespan in a variety of model organisms. However, restriction of the essential amino acid methionine can produce similar physiological effects to CR without reducing caloric intake. Like CR, methionine restriction (MR) extends longevity, reduces oxidative stress, and improves metabolism. While CR and MR share physiological similarities, there is growing evidence of disparate molecular mechanisms between the two interventions. The side chain of methionine contains sulfur suggesting a potential importance of sulfur redox in MR. This sulfur is easily oxidized to form methionine sulfoxide which is potentially detrimental to protein function. Eukaryotic cells have evolved methionine sulfoxide reductase A (MsrA) as an enzymatic means to repair this oxidation. Here, we questioned whether methionine oxidation, mediated by MsrA, may play an important role in the physiological outcomes of MR by testing this intervention in mice lacking MsrA (MsrA KO). Under MR, MsrA KO mice experience reduced body weight and fat as expected, and also experience improved glucose tolerance as a functional outcome. However, other metabolic markers including O2 consumption and CO2 production in MsrA KO mice were unaffected by MR which contrasts the increase observed in wild type MR mice. Moreover, we noted significant differences between MsrA KO and wild type mice under MR in other markers of healthspan such as activity. These results suggest that methionine oxidation and/or MsrA play important roles in at least some of the mechanistic outcomes mediated by MR.

*Correspondence to: Kevin Thyne, Barshop Institute, UTHSCSA, TX. Email: thyne@livemail.uthscsa.edu

## Cerebrovascular deficits in mouse models of tauopathy are associated with nitric oxide synthase dysfunction

Candice E. Van Skike^1,^*, Stacy A. Hussong^1,2^, Stephen F. Hernandez^1^, and Veronica Galvan^1,2^

^1^Barshop Institute for Aging and Longevity Studies and the Department of Cellular and Integrative Physiology, UTHSCSA, TX, USA. ^2^Department of Veterans Affairs, STVHCS at San Antonio, TX, USA

Our lab has recently reported the accumulation of tau oligomers in the cerebral microvasculature of human patients with tauopathies, including Alzheimer’s disease (AD), dementia with Lewy bodies, and progressive supranuclear palsy, as well as in the Tg2576 (hAPPswe) mouse model of AD. However, the functional consequences of cerebrovascular tau accumulation have yet to be determined. We assessed the coupling between increased neural activity and increased cerebral blood flow, which is a highly regulated processes called neurovascular coupling (NVC). Therefore, the aim of the present study was to establish cerebrovascular dysfunction in several different models of tauopathy. Laser Doppler flowmetery was used to measure the increase in cerebral blood flow in response to whisker stimulation, an indicator of NVC, in the following mouse models: JNPL3 (P301L), PS19 (P301S), and hTau mice compared to appropriate WT controls for each strain. At seven months, all transgenic mice exhibited neurovascular coupling deficits compared to WT controls. We also assessed endothelium-dependent vasodilation in vivo in the PS19 (P301S) and hTau mice. The tau transgenic mice showed reduced evoked CBF in response to the endothelium-dependent vasodilator, acetylcholine compared to WT controls. Both NVC and endothelium-dependent vasodilation are dependent on nitric oxide synthase activity. In order to assess the impact of tau oligomers on nitric oxide synthase function, we treated neurons or endothelial cells with oligomeric tau and measured the relative phosphorylated and total protein concentration of nNOS and eNOS from neurons and endothelial cells, respectively. Our results indicate that oligomeric tau inhibits the activation of both nNOS and eNOS, which may underlie the cerebrovascular dysfunction observed in these models of tauopathy. These data suggest a link between cerebrovascular function and tauopathy. It is not yet clear whether impaired cerebrovascular function is a cause or consequence of tauopathy, particularly with regards to the accumulation of soluble tau aggregates within the cerebrovasculature.

*Correspondence to: Candice E. Van Skike, Barshop Institute, UTHCSA, TX, USA. Email: vanskike@uthscsa.edu

##  Funding

CVS: Alzheimer’s Association AARF-17–504,221; SFH: NIH R25NS080684; VG: 1R01AG057964-01, 1-I01-BX002211-01 VA Merit Review Award, Owens Foundation, Bailey Family Alzheimer’s Fund.

## Phytoecdysteroids accelerate recovery of skeletal muscle function following in vivo eccentric contraction-induced injury in young and old mice

Kevin A. Zwetsloot^1,^*, Charles F. Hodgman^1^, Joshua S. Godwin^1^, R. Andrew Shanely^1^

^1^Department of Health & Exercise Science; Integrated Muscle Physiology Laboratory, Appalachian State University at Boone, NC, USA

Phytoecdysteroids are natural plant steroids synthesized by a variety of hardy plants. Previous work by our group has shown that administration of phytoecdysteroids, such as 20hydroxyecdysone (20E), in old mice can lead to an increase in protein synthesis signaling and skeletal muscle fiber size. To investigate whether phytoecdysteroids enhance skeletal muscle recovery from eccentric contraction-induced damage, young (6.1 ± 0.4 mo) and old (26.5 ± 0.5 mo) mice were subjected to injurious eccentric contractions (EC), followed by 7 days of 20E supplementation or placebo (PLA). Mice were anesthetized with isoflurane and then in vivo isometric contractions were performed (Aurora Scientific, 1300A) to obtain torque-frequency relationships (TF) of the anterior crural muscle group (PRE), followed by 150 EC. Following recovery from anesthesia, the mice received either 20E (50 mg•kg-1 BW) or PLA (saline) by oral gavage. Mice were gavaged daily for 6 days and on day 7, TF was re-assessed (7-day). Significant decreases in TF in young and old mice were measured immediately after EC (both p < 0.001) and PLA groups remained depressed at day 7 (PRE vs 7-day; Young p = 0.048; Old p < 0.001). However, 20E supplementation completely recovered TF after 7 days in both young and old mice (PRE vs. 7-day; Young p = 0.396 & Old p = 0.383). These findings suggest that 20E may exert anabolic or ergogenic effects in skeletal muscle of male mice that accelerate recovery of in vivo isometric skeletal muscle function following EC-induced injury.

*Correspondence to: Kevin A. Department of Health & Exercise Science; Integrated Muscle Physiology Laboratory, Appalachian State University at Boone, NC, USA. Email: vanskike@uthscsa.edu

